# Catch-up growth up to ten years of age in children born very preterm or with very low birth weight

**DOI:** 10.1186/1471-2431-5-26

**Published:** 2005-07-20

**Authors:** Noël BB Knops, Kommer CA Sneeuw, Ronald Brand, Elysee TM Hille, A Lya den Ouden, Jan-Maarten Wit, S Pauline Verloove-Vanhorick

**Affiliations:** 1 Department of Paediatrics; Leiden University Medical Center, J6S; P.O. Box 2600; 2300 RC Leiden; The Netherlands; 2 TNO Prevention and Health, Child Health Division, Leiden; The Netherlands; 3 Department of Medical Statistics, Leiden University Medical Center; The Netherlands

## Abstract

**Background:**

Improved survival due to advances in neonatal care has brought issues such as postnatal growth and development more to the focus of our attention. Most studies report stunting in children born very preterm and/or small for gestational age. In this article we study the growth pattern of these children and aim to identify factors associated with postnatal catch-up growth.

**Methods:**

1338 children born with a gestational age <32 weeks and/or a birth weight of <1500 grams were followed during a Dutch nationwide prospective study (POPS). Subgroups were classified as appropriate for gestational age and <32 weeks (AGA) or small for gestational age (<32 wks SGA and ≥32 wks SGA). Data were collected at different intervals from birth until 10 years for the 962 survivors and compared to reference values. The correlation between several factors and growth was analysed.

**Results:**

At 10 years the AGA children had attained normal height, whereas the SGA group demonstrated stunting, even after correction for target height (AGA: 0.0 SDS; SGA <32 wks: -0.29SDS and ≥32 wks: -0.13SDS). Catch-up growth was especially seen in the SGA children with a fast initial weight gain. BMI was approximately 1 SD below the population reference mean.

**Conclusion:**

At 10 years of age, children born very preterm AGA show no stunting. However, many children born SGA, especially the very preterm, show persistent stunting. Early weight gain seems an important prognostic factor in predicting childhood growth.

## Background

Advances in neonatal care in the past two decades have improved survival of very preterm and low birth weight infants dramatically. This has brought issues such as developmental outcome and growth of these 'survivors' to the focus of our attention [1]. In 1989 Barker et al demonstrated an increase in mortality from coronary heart disease in adulthood in subjects who had low birth weight [2], and this observation was followed by studies of many others on long term adverse effects of intra-uterine growth retardation [3]. Furthermore not only the appropriateness of weight for gestational age but the course of postnatal growth seems to predict later neurodevelopmental outcome in children with low birthweight [4]. Follow-up of these children is an important tool in learning to understand and tackling problems that can occur.

Several studies have studied physical growth during early childhood in children born preterm, small for gestational age (SGA) or appropriate for gestational age (AGA). They show stunting for all groups with limited to no catch-up growth especially in SGA group [5-11]. This could not be explained by factors known to influence postnatal growth such as: hospitalisation, interventional status, bronchopulmonary dysplasia, socio-economic status, parental level of education, neonatal thyroxine or thyroxine stimulating hormone levels [8,12].

Most data reporting on further growth into adolescence show incomplete catch up growth and therefore persistent stunting, especially in those born SGA [13-16]. Those who report attainment of normal height studied mainly children born AGA [17,18]. Only the female SGA cohort followed by Hack et al reached normal adult height but their male counterparts did not [19]. Thus stunting is primarily seen in the infant with low birth weight for gestational age. These findings suggest a long-term relationship between intrauterine growth retardation and growth into adolescence. Determining the factors that cause growth impairment in these children may enable us to find therapeutic or preventive options.

Following an earlier report on height achievement at five years of a large cohort of children born very preterm or VLBW [12], we studied the growth pattern up to 10 years of age in the same children. The data were collected in a nationwide effort of Dutch pediatricians: the " Project On Preterm and Small for Gestational Age Infants in The Netherlands" (POPS) [20]. We aimed to identify factors associated with catch-up growth between 5 and 10 years of age. Predictive values and sensitivity rates of short stature at younger age for having short stature at 10 years of age were determined. Finally, Body Mass Index was calculated to assess nutritional status in our study population.

## Methods

The POPS study was started in 1983 to investigate relationships between perinatal factors, mortality and morbidity in very preterm and VLBW infants. The protocol was approved by the medical ethics committees of the participating hospitals and parents gave informed consent. The study population consisted of live-born infants in The Netherlands from January 1 to December 31, 1983, with a gestational age of <32 weeks and / or a birth weight of <1500 g. Follow-up examinations were performed at the approximate corrected ages of: 3, 6, 12 and 24 months and again at the approximate chronological age of 5 and 10 years. Of the original 1338 children, 962 were alive at 10 years of age.

Paediatricians performed length/height measurements at 3 and 6 months and at 1, 2 and 5 years. At 10 years, the parents reported height as part of an extensive questionnaire. Recent growth curves were used as reference values for body length/height measurements and body mass index [21,22]. These measurements were collected in 1996–1997 during a nationwide growth study on individuals of Dutch origin aged 0 to 21 years. Based on these reference values, for each child the standard deviation score (SDS) was calculated using corrected age (height minus age/sex-specific mean height in the reference population divided by age/sex-specific SD in the reference population). A child was considered having "short stature" at a specific age if his/her height was below the 10th percentile (being equivalent to approximately -1.3 SDS) of the reference population.

Since in our earlier report [12] reference values were obtained from the Dutch growth curves of 1980 [23], the SD-scores employed in the current study were also compared to those based on the 1980 reference data. Only marginal differences were observed, with the SD-scores based on the 1996–1997 reference values being on average 0.04 SD lower than those based on the 1980 reference data (range -0.37 to 0.34).

Classification of AGA/SGA (appropriate/small-for-gestational-age) infants was based on measurements of birth weight relative to gestational age, described by Kloosterman [24,25]. An SGA infant has a birth weight less than the 10th percentile for gestational age, gender and parity.

For the present study, non-Caucasian children and children with congenital malformations were excluded, since the reference data cannot be used for these children. Hence, 753 of the 962 surviving children were included in the analysis.

Statistical analyses included the following steps. The growth pattern of the children over time was examined by comparing mean length/height SDS, median percentiles and percentages of children below the 10th percentile (<P10) at the various ages. Considering possible bias due to differences in genetic growth potential, target height (TH) was calculated for each individual using parental height data compared to Dutch national growth statistics. Target height was defined as the mean of parental height corrected for the mean difference in height between sexes, plus the mean increase in height per generation (according to Dutch reference values [23]:((height father + height mother plus or minus 12 cm) divided by 2 + 3 cm). Using individual target height, target height SD-scores (TH-SDS) and height SDS corrected for TH (HSDS_corr_) were assessed (TH-SDS: (TH minus mean adult height (male or female)) divided by the sex-specific standard deviation for adult height) and HSDS_corr_: height-SDS minus TH-SDS). Separate analysis of growth patterns was performed among three subgroups of children: very preterm but appropriate-for-gestational age (<32 wks/AGA), very preterm and small-for-gestational age (<32 wks/SGA) and gestational age of 32 weeks or over who were small-for-gestational age (≥32 wks/SGA). The clinical characteristics of these subgroups are shown in table 1. The relationship between several factors and catch-up growth between 5 and 10 years of age was explored by means of regression analysis. Differences between the 5- and 10-years height SDS were calculated and compared between the three defined subgroups, as well as for single/multiple birth, sex, socio-economic status, maternal and paternal height. Cross-tabulations were used to examine the predictive values and sensitivity rates of height <P10 (and <P3) at various ages for height <P10 (and <P3) at 10 years of age. Follow-up data on weight were used to study the effect of early postnatal growth on height at 10 years and to assess BMI.

**Table 1:** Clinical characteristics of the different subgroups according to gestational age and weight of the entire study population, of the group lost to follow up at age10 years and of the children with available data on weight increase during the first three months.

		Sex	Mean Gestational Age	Mean Birth-weight	Multiple Birth	Respiratory support >1 week	Intra Cranial Haemorrhage	Necrotising Entero Colitis	Mean Hospital stay
	n	(% males)	(wk)	(gram)	(%)	(%)	(%)	(%)	days
<32 wks/AGA	445	53.1	29.7	1412	26.7	27.8	21.7	4.5	64
<32 wks/SGA	86	51.2	30.3	968	11.6	23.3	14.0	8.1	86
≥32 wks/SGA	222	49.5	34.5	1278	16.7	2.5	5.1	5.6	59
Lost to follow up at age 10 years									
	243	49.2	31.1	1326	23.0	18.4	18.3	5.8	63
Weight Increase at 3 months									
<3500 gr	257	45.3	32.1	1275	21.7	20.6	14.9	6.2	69
≥3500 gr	389	54.6	30.1	1349	21.2	19.3	16.3	4.6	62

## Results

Of the 753 children in the POPS cohort who met our selection criteria, length/height measurements were available for 649 to 721 children at various ages up to 5 years of age (86% to 96%). Table 1 shows the clinical characteristics of the survivors according to subgroup. At 10 years, height data were obtained for 510 children (68%).

Table 2 shows the mean length/height data expressed as SDS, median percentiles and proportions of children <P10 for the total and subgroups. In the total group there was a substantial increase in mean SDS from -1.28 at 3 months to -0.18 at 10 years of age, similarly reflected in an increase in median percentile and decrease in percentage of children <P10. Both the median percentile and the percentage <P10 at 10 years are similar to those at 5 years of age. The 17% of children <P10 at 10 years of age were on average 13 cm shorter (range 8 to 25 cm) than sex/age-specific reference values (131 cm versus 144 cm).

**Table 2:** Growth measurements uncorrected and corrected for target height (HSDS_corr_) at various ages for subgroups based on weight for gestational age

	Length/height-SDS		Percentiles	HSDS_corr_	
Approximate age	*n*	mean	<P10	*n*	SDS
Total group					
3 months	*659*	-1.28	48%	*633*	-1.11
6 months	*649*	-0.95	39%	*626*	-0.76
1 year	*670*	-0.76	32%	*649*	-0.58
2 years	*651*	-0.64	28%	*628*	-0.44
5 years	*721*	-0.18	18%	*719*	0.03
10 years (observed)^1^	*510*	-0.18	17%	*509*	-0.07
*10 years (estimated)*^2^	*723*	*-0.24*	*17%*	*721*	*-0.09*
					
<32 wks/AGA					
3 months	*388*	-0.68	29%	*381*	-0.58
6 months	*383*	-0.49	24%	*382*	-0.39
1 year	*387*	-0.43	22%	*386*	-0.34
2 years	*378*	-0.33	20%	*374*	-0.21
5 years	*422*	0.11	7%	*420*	0.18
10 years (observed)^1^	*298*	-0.04	15%	*298*	0.00
*10 years (estimated)*^2^	*423*	*-0.06*	*15%*	*422*	*0.01*
					
<32 wks/SGA					
3 months	*78*	-2.22	82%	*78*	-1.90
6 months	*72*	-1.72	64%	*72*	-1.39
1 year	*78*	-1.38	46%	*78*	-1.08
2 years	*78*	-1.33	45%	*78*	-1.02
5 years	*85*	-0.65	24%	*85*	-0.37
10 years (observed)^1^	*64*	-0.55	25%	*64*	-0.29
*10 years (estimated)*^2^	*85*	*-0.64*	*24%*	*85*	*-0.33*
					
≥32 wks/SGA					
3 months	*193*	-2.19	78%	*174*	-1.90
6 months	*194*	-1.60	72%	*172*	-1.31
1 year	*205*	-1.16	48%	*185*	-0.87
2 years	*195*	-0.95	36%	*176*	-0.67
5 years	*214*	-0.62	25%	*214*	-0.35
10 years (observed)^1^	*148*	-0.35	17%	*147*	-0.13
*10 years (estimated)*^2^	*215*	*-0.48*	*16%*	*214*	*-0.20*

^1 ^observed length for availabe cases

^2 ^observed length for availabe cases + estimated length (based on length at 5 years) for missing cases

To examine whether the missing data pattern at 10 years was random or not, mean SD-scores at 5 years (*n *= 721) were compared between the 508 children for whom height measurements were available at both 5 and 10 years and the 213 children without a 10-years height measurement. A statistically significant difference was noted (*p *= 0.02), with mean SD-scores of -0.18 and -0.63 for those with and without 10-years data, respectively. To adjust for possible bias due to drop-out of shorter children, SD-scores for the 213 children without height measurements at 10 years but for whom height at 5 years was available were estimated by means of a linear regression equation. This equation was based on regression of 10-years SDS on those at 5 years in the subgroup of 508 children with both measurements available: 10-years SDS = 0.006 + (0.832 × 5-years SDS); explained variance *R*^2 ^= 0.58. As shown in Table 2, this calculation yielded a mean height SDS of -0.24 (instead of -0.18 for available measurements).

For the <32 wks/AGA children moderate stunting was noted with catch-up growth up to 5 years of age but none between age 5 and 10 years. For both the <32 wks/SGA and the ≥32 wks/SGA children, more serious stunting was noted with continuing catch-up growth up to 10 years.

The <32 wks/AGA group had a normal target height (-0.01 SDS). However, both subgroups born SGA had lower target height of -0.29 SDS. Therefore, in the SGA group length/height-SDS corrected for TH (HSDS_corr_) is substantially higher than uncorrected length/height-SDS (mean difference HSDS_corr _- length/height-SDS: + 0.30 SDS in the <32 weeks and +0.27 SDS in the ≥32 weeks group). Correction in the <32 wks/AGA group was small (mean difference: +0.10 SDS).

The relationship between several other factors and catch-up growth between 5 and 10 years of age was examined among those children for whom height measurements were available at both ages. The change between the 5- and 10-years SD-scores for height was on average 0.07 SDS, a large range was noted at the individual level (-2.96 to 3.74). Although parental height and, to a smaller extent, multiple birth and socio-economic status were significantly associated with height at 5 and 10 years, these factors were not associated with catch-up growth in that period (respectively p = 0.14 for multiple birth and p = 0.3 for socio-economic status). Regression analysis showed that, in addition to the observed differences in catch-up growth between the three subgroups (*p *= 0.001), the level of catch-up growth was independently associated with sex (*p *= 0.03). Table 3 shows that more catch-up growth was observed for boys than for girls. Regarding the different subgroups, more substantial catch-up growth was observed for the SGA boys, especially for those of ≥32 weeks (mean SD-score change of 0.37). Only marginal changes in 5- and 10-years mean SD-scores were seen for girls in all three subgroups, as well as for the <32 wks/AGA boys. In these children, the percentage of children <P10 at 10 years of age were even slightly higher than at 5 years of age. The highest percentage <P10 at 10 years of age was observed for <32 wks/SGA boys, in spite of a significant decrease in the last 5 years (from 49% to 36%).

**Table 3:** Comparison of growth patterns in boys and girls in the different subgroups

		Mean length/height SD-scores			Proportions < P10	
	*n*	5 yr	10 yr	change	5 yr	10 yr
<32 wks/AGA						
girls	*133*	-0.05	-0.15	-0.10	9%	14%
boys	*164*	-0.24	-0.21	0.03	17%	20%
						
<32 wks/SGA						
girls	*31*	-0.57	-0.50	0.07	19%	26%
boys	*33*	-1.14	-0.89	0.25	49%	36%
						
≥32 wks/SGA						
girls	*66*	-0.69	-0.52	0.17	21%	24%
boys	*63*	-0.72	-0.35	0.37	25%	19%

The predictive value of height <P10 at various ages for height <P10 at 10 years was examined by cross-tabulation of available measurements. The predictive value at 5 years of age was 69% (Table 4). Lowering the cut-off point from P10 to P3, a stricter criterion for "short stature", did not increase the predictive value.

**Table 4:** Relation between short stature at age 5 and 10 years

	n	At 10 years	
		<P10	≥P10
At 5 years			
<P10	*95*	66 (69%)	29 (31%)*
≥P10	*413*	37 (9%)	376 (91%)
			
		<P3	≥P3

At 5 years			
<P3	*44*	29 (66%)	15 (34%)†
≥P3	*464*	18 (4%)	446 (96%)

* median percentile: 23 (16 children < P25)

† median percentile: 14 (12 children < P25)

To examine the effect of early postnatal growth on height at 10 years we looked at the average increase in weight during the first three months, approximately 3500 grams, and used this as a cut-off point defining two subgroups (fast and slow initial growth rate). Clinical characteristics and HSDS_corr _for these groups are given in table 1 and 5, respectively. Children with fast initial growth already attained normal height for TH SDS at 5 years, while those with slow initial growth showed persistent stunting. The fast subgroup of <32 wks/AGA attained full catch-up growth at 2 years of age. The fast subgroup of the ≥32/SGA weeks did so at age 5 year. All slow equivalents show persistent stunting. Despite initial high catch-up growth in the fast subgroup of the very preterm SGA they remain stunted at age 10 years.

**Table 5:** Height corrected for target height (HSDS_corr_) in children with fast and slow initial growth for the different subgroups based on weight for gestational age.

	HSDS_corr_			
	Weight increase at 3 months			
	<3500 gram		≥3500 gram	
Approximate age	*n*	mean	*n*	mean
Total group				
3 months	*257*	-1.85	*389*	-0.59
6 months	*252*	-1.41	*371*	-0.32
1 year	*263*	-0.99	*370*	-0.27
2 years	*243*	-0.85	*367*	-0.18
5 years	*271*	-0.61	*391*	-0.08
10 years (observed)	*189*	-0.37	*282*	-0.08
*10 years (estimated)*	*270*	-0.42	*392*	-0.08
				
<32 wks/AGA				
3 months	*100*	-1.29	*278*	-0.32
6 months	*101*	-1.05	*263*	-0.14
1 year	*104*	-0.75	*262*	-0.19
2 years	*98*	-0.65	*258*	-0.08
5 years	*108*	-0.41	*278*	-0.04
10 years (observed)	*77*	-0.35	*199*	-0.07
*10 years (estimated)*	*109*	-0.33	*279*	-0.07
				
<32 wks/SGA				
3 months	*37*	-2.47	*41*	-1.38
6 months	*34*	-2.03	*37*	-0.82
1 year	*38*	-1.36	*38*	-0.81
2 years	*38*	-1.34	*38*	-0.64
5 years	*41*	-0.91	*41*	-0.37
10 years (observed)	*29*	-0.46	*32*	-0.37
*10 years (estimated)*	*41*	-0.60	*41*	-0.33
				
≥32 wks/SGA				
3 months	*120*	-2.20	*70*	-1.30
6 months	*117*	-1.57	*71*	-0.78
1 year	*121*	-1.11	*70*	-0.31
2 years	*107*	-0.85	*71*	-0.28
5 years	*122*	-0.73	*72*	-0.06
10 years (observed)	*83*	-0.38	*51*	-0.06
*10 years (estimated)*	*120*	-0.44	*72*	-0.04

Body Mass Index (BMI) and BMI-SDS for the total group and different subgroups was calculated. BMI for all the children was approximately 1 kg/m2 below Dutch reference values [22]. However, the overall trend is similar to Dutch reference values. Mean BMI-SDS in the total group was -0.73 at 10 years. The SGA group had the lowest BMI (mean BMI-SDS: -1.02 and -0.96 for the <32 weeks and ≥32 weeks, respectively). The <32 wks/AGA had the highest BMI (-0.56 SDS). BMI-SDS was lower in early childhood in both the SGA and AGA group.

## Discussion

Our results demonstrate, despite catch-up growth, persistent stunting until the age of 10 year in children born SGA, both before and after 32 weeks. The <32 wks/AGA showed little to no stunting and no catch-up growth from 5 to 10 yr. The improvement of SDS does not necessarily implicate a decrease in the absolute difference of a subject's height versus the average for age so that catch-up growth can remain unnoticed for the families. We are aware that the reporting by the parents of their child's height at 10 yrs could be imprecise, however this will be the same for all groups and have therefore no effect on the final conclusion of this study. Correction for Target Height suggests that the low height SDS in SGA children is partially associated with genetic factors. Failing to correct for target height leads to an overestimating of stunting in especially the SGA children (±0.3 SDS). This point was also made by Ford et al [26]. These data show that intrauterine growth retardation has more important long-term impact on growth than gestational age, suggesting an intrinsic lesser growth potential in children born small for date or a persistent effect of growth retardation in utero. A study by Sung et al showed more stunting in the SGA at 4 yr. compared to weight matched children born AGA (mean gestational age: SGA: 29 wk; AGA 26 wk) although the latter had more complications in early infancy [10]. Peralta-Carcelen et al demonstrated persistent stunting in adolescence in extremely low birth weight children without major handicaps [16]. Also in our study there was no significant difference in the incidence of neonatal morbidity between the subgroups (data published earlier [27]). Further analysis of the extent of each complication as stated in table 1 was not performed in this study but could be important for growth in individual cases (for example gut resection after NEC). Thus, intrauterine growth retardation seems a more important factor in determining childhood growth than neonatal complications associated with long-term physical and mental impairments.

Early recognition of persistent stunting can be of value for better treatment and preventive measures. In our study the predictive value of height <P10 at various ages for height at 10 years increased with age until 69% at 5 years. Lowering the cut-off point to <P3 did not improve the predictive value much, probably because of continuing catch-up growth. If in the future these cut-off points are used as a criterion to start growth enhancing treatment at an age of 5 years, the results of treatment should be compared with these figures on spontaneous growth.

Early postnatal (catch-up) growth possibly provides a useful tool for predicting height at 10 years. Because of difficulty in the accurate measurement of length at birth we did not register length at birth in our study and expressed early catch-up growth in weight gain. The arbitrary cut-off point we chose of the mean weight increase in the first 3 months (approximately 3500 grams) could result in wrongfully selecting children to a group with a different growth potential compared to their own. This would result in pulling height outcome of the different groups toward each other (regression to the mean). However our data show a clear difference between the subgroups, supporting the possibility of an inherently different growth potential. Children with an initial high catch-up growth generally attain normal height but the slow starters and the very preterm SGA do not. A similar observation was made by others [28,29]. Fewtrell et al suggested a distinction in children with an intrinsic or extrinsic disturbed intra-uterine growth potential after the observation that preterm children whose mothers had hypertension or toxemia showed more catch-up growth and less stunting at 12 year compared to those whose mothers were unaffected [30].

Variation in early catch-up could be caused by a disturbance in hormonal response. Albertsson-Wikland et al demonstrated low growth hormone, IGF-I, IGFBP-3 and leptin secretion in the subgroup of term SGA children with little catch-up growth at two months and short final height versus term SGA children with good catch-up and no stunting [31]. This could be due to rare IGF-I polymorphisms more frequently seen in the SGA and is associated with low bone mineral density [32]. This again could be a possible explanation for the high alkaline phosphatase levels in especially SGA preterm infants who remain short at age 12 yr, demonstrated by Fewtrell et al who suggested early metabolic bone disease as a cause for stunting in these children [30].

Preterm boys demonstrated more stunting at age 5 and 10 yr. but also showed more catch-up growth than girls. Only the ≥32 wks/SGA boys demonstrated less stunting than the girls at age 10 years. Other studies also demonstrated more stunting in SGA boys [19,33]. Possible explanations for the higher catch-up growth in boys in our study include a higher degree of stunting compared to the girls (which leaves them more to catch-up), a higher morbidity in the neonatal period and later life, and a different time of onset of puberty. Girls born SGA were shown to have an exaggerated adrenarche by Ibanez et al [34,35] but no difference has been demonstrated in the onset of puberty between very preterm SGA and a control population [14,26,36]. Nonetheless an advanced bone age has been reported despite similar sexual maturation in adolescents of very preterm SGA origin [16,37]. Advanced bone age could further compromise final height in these children. Unfortunately, we have no data on sexual maturity in our population.

Our study population demonstrated a low mean BMI compared to their peers. However at age 10 years there was a slight increase in BMI-SDS in relation to the previous ages. Although BMI-SDS is lowest in the SGA, a similar trend is observed in all groups. Saigal et al [14] demonstrated a 1.9 SD lower BMI in their very preterm/ SGA group vs. controls at age 8. At adolescence there remained a difference of -1.5 SD in BMI. A cohort study from the United States performed by Hack et al showed eventual normal adult BMI in SGA girls (0.4 SDS) but not in boys (-0.3 SDS) [19]. Hediger et al demonstrated lower mid-upper arm circumference in SGA children (-0.5 SDS) [38]. These data suggest a diminished nutritional status again especially in the SGA group when compared to their peers. This does not necessarily implicate a higher incidence of wasting in the study population but could also be explained by the increased incidence of obesity in the Dutch children and higher BMI reference values of 1997 compared to 1980.

## Conclusion

In this study we show that children born very preterm with an appropriate weight for gestational age show little or no stunting at the age of 10 yr. However children born small for gestational age, especially those born <32 weeks, show persistent stunting at age 10 yr notwithstanding considerable catch-up growth in many of them. Early weight gain seems an important factor in predicting catch-up growth. Knowledge about spontaneous growth is useful if one considers offering growth-enhancing treatment to low birth weight children with short stature in early childhood.

## List of abbreviations

SGA Small for Gestational Age

AGA Appropriate for Gestational Age

SDS Standard Deviation Score

TH Target Height

HSDS_corr _Height corrected for Target Height

BMI Body Mass Index

## Competing interests

The author(s) declare that they have no competing interests.

## Authors' contributions

NK and KS were involved in the design of the analysis and in writing the manuscript. RB performed the statistical analyses. EH and LdO provided the data of the POPS cohort. JMW supervised the design and writing process. SVV is projectleader of POPS. All authors contributed to the preparation of the manuscript.

## Pre-publication history

The pre-publication history for this paper can be accessed here:


